# *LbMYB48* positively regulates salt gland development of *Limonium bicolor* and salt tolerance of plants

**DOI:** 10.3389/fpls.2022.1039984

**Published:** 2022-10-26

**Authors:** Guoliang Han, Ziqi Qiao, Yuxia Li, Zongran Yang, Ziwei Zhang, Yuanyuan Zhang, Jinjiao Guo, Lili Liu, Chengfeng Wang, Baoshan Wang

**Affiliations:** Shandong Provincial Key Laboratory of Plant Stress Research, College of Life Sciences, Shandong Normal University, Shandong, China

**Keywords:** *limonium bicolor*, salt gland, salt secretion, MYB transcription factor, salt tolerance

## Abstract

*Limonium bicolor* is a dicotyledonous recretohalophyte with several multicellular salt glands on the leaves. The plant can directly secrete excess salt onto the leaf surface through the salt glands to maintain ion homeostasis under salt stress. Therefore, it is of great significance to study the functions of genes related to salt gland development and salt tolerance. In this study, an R1-type MYB transcription factor gene was screened from *L. bicolor*, named *LbMYB48*, and its expression was strongly induced by salt stress. Subcellular localization analysis showed that *LbMYB48* was localized in the nucleus. LbMYB48 protein has transcriptional activation activity shown by transcriptional activation experiments. The density of salt glands in the leaves and the salt secretion capacity of *LbMYB48*-silenced lines were decremented, as demonstrated by the leaf disc method to detect sodium ion secretion. Furthermore, salt stress index experiments revealed that the ability of *LbMYB48*-silenced lines to resist salt stress was significantly reduced. *LbMYB48* regulates salt gland development and salt tolerance in *L. bicolor* mainly by regulating the expression of epidermal cell development related genes such as *LbCPC-like* and *LbDIS3* and salt stress-related genes (*LbSOSs*, *LbRLKs*, and *LbGSTs*) as demonstrated by RNA-seq analysis of LbMYB48-silenced lines. The heterologous over-expression of *LbMYB48* in *Arabidopsis thaliana* improves salt tolerance of plants by stabilizing ion and osmotic balance and is likely to be involved in the abscisic acid signaling pathway. Therefore, *LbMYB48*, a transcriptional activator regulates the salt gland development of *L. bicolor* and salt tolerance of *L. bicolor* and *A. thaliana.*

## Introduction

Soil salinization is a process of land degradation, leading to the excessive accumulation of soluble salt in soil (Ayub et al., 2020; Savich et al., 2021). Moreover, about 20% of cultivated land and 33% of irrigated land globally have been affected and degraded by soil salinization to varying degrees. Soil salinization has become one of the primary environmental and socio-economic problems in the world (Shrivastava and Kumar, 2015; Hassani et al., 2021). Most crops are salt-sensitive plants, and their salt tolerance is up to 50 mM concentration of sodium ions (Na^+^) in their growth environment. The excessive accumulation of Na^+^ in plants may exceed their salt tolerance and have a seriously adverse impact on the growth and development of plants (Flowers et al., 2010; Litalien and Zeeb, 2020). However, halophytes can survive and complete their life cycles in environments containing at least 200 mM concentrations of sodium chloride (NaCl) (Flowers and Colmer, 2008; Flowers and Colmer, 2015).

*L. bicolor* is a typical dicotyledonous recretohalophyte (Yuan et al., 2014; Li et al., 2020). The leaf surface of *L. bicolor* possesses a unique epidermal structure called a salt gland, which can secrete excess salt onto the leaf surface and reduce the damage of salt stress to the plant (Yuan et al., 2022). In 1990, Wiehe and Breckle proposed the development mechanism model of a multicellular salt gland of *L. bicolor*. It was assumed that the salt gland is a sixteen-cell complex formed by five successive divisions of a protoepidermal cell (Han et al., 2022). This complex comprises four secretory cells, four adjacent cells, four inner cup cells, and four outer cup cells (Wiehe and Breckle, 1990). Previous studies exhibited that a large number of salt crystals were present on the leaf surface of *L. bicolor* grown in a salted environment and were concentrated in the secretory pores of salt glands, as confirmed by a scanning electron microscope (Feng et al., 2014). Furthermore, the morphological changes and salt secretion rate of salt glands can be quantified by the leaf disc secretion method, and the secretory capacity of a single salt gland can be determined by the collection of secretory fluid on the leaf surface and the determination of ion concentration (Feng et al., 2014; Deng et al., 2015). Further, the virus-induced gene silencing (VIGS) method can be efficiently applied for gene silencing in *L. bicolor* to study the development and salt secretion mechanism of the salt gland (Lu et al., 2020).

In recent years, plant MYB transcription factors are a popular class of transcription factors related to the regulation of plant growth and development, physiological metabolism, cell morphology, and abiotic stress resistance (Wang et al., 2021a). They are common in plants and are one of the largest transcription factor families in plants (Dubos et al., 2010; Jiang and Rao, 2020). MYB transcription factors such as GLABRA1 (GL1), MYB23, and CAPRICE (CPC) are involved in epidermal cell development for plant growth. Further, OsMYB2 can positively regulate a variety of abiotic stresses by accumulating more soluble sugars and proline for plant tolerance to abiotic stresses (Yang et al., 2012); AtMYB20 can enhance abscisic acid (ABA) signaling and positively regulate plant salt tolerance by inhibiting the expression of ABA signaling negative regulators such as ABA INSENSITIVE 1 (ABI1), ABI2 and protein phosphatase 2C (PP2C) (Cui et al., 2013); *ZmMYB48* and *OsMYB48-1* can positively regulate the expression of ABA signaling positive regulators to enhance ABA signaling and positively regulate drought tolerance of plants (Xiong et al., 2014; Wang et al., 2017).

In this research, a gene encoding an R1-MYB transcription factor in *L. bicolor* was identified, which was highly expressed under salt treatment and may be involved in the development of the salt gland, and salt tolerance in *L. bicolor*. The gene was named *LbMYB48* as it shares some homology with MYB48 proteins in the plant. Further, the bioinformatics, subcellular localization, and transcriptional activation activity of LbMYB48 were determined. The function of *LbMYB48* in salt gland development and salt tolerance of *L. bicolor* was detected by VIGS technology, leaf disc method, physiological index determination, and RNA-seq experiments. In addition, the salt tolerance of *LbMYB48* heterologous over-expression lines in *A. thaliana* was also determined by a range of physiological and molecular indicators.

## Materials and methods

### Plant materials and growth conditions

*L. bicolor* seeds were collected from the saline-alkali land of the Yellow River Delta in Dongying, Shandong, China, and were stored in a 2 °C freezer until further use. The seeds were soaked in water for one day (d) before planting in nutrient soil and placed in an artificial climate chamber, where the temperature was set at 25 °C d/22 °C night with the relative humidity set at 75%. The light intensity was set at 160 μmol/m^2^/s, and the photoperiod was set at 16 h light/8 h dark. VIGS silencing treatment was performed when *L. bicolor* plants were grown to the six-leaf stage (Lu et al., 2020). The ecotype Columbia-0 (Col-0) and non-transgenic Col-0 *A. thaliana* were used as wild-type control for the transgenesis. The artificial climate chamber used to culture *A. thaliana* was set at 22 °C d/night 18 °C, 150 μmol/m^2^/s light intensity, 16 h light/8 h dark the photoperiod, and 75% the relative humidity (Norén et al., 2004; Liu et al., 2019).

### Bioinformatic analysis of LbMYB48

The full-length *LbMYB48* sequence was amplified from the cDNA of *L. bicolor*. The sequence alignment was performed using DNAMAN (version 7.0), and the conserved domains of *LbMYB48* were analyzed using SMART software (http://smart.embl-heidelberg.de/). A phylogenetic tree of *LbMYB48* was constructed using MEGA software (version 5.0), and the promoter *cis*-acting elements of *LbMYB48* were analyzed using Plant CARE software (http://bioinformatics.psb.ugent.be/webtools/plantcare/html/) (Lescot et al., 2002; Yuan et al., 2019).

### Subcellular localization of LbMYB48

The open reading frame (ORF) of *LbMYB48* was specifically amplified by PCR reaction with forward and reverse primers, taking the cDNA library of *L. bicolor* as the template (**Supplementary Table 1**). The pCAMBIA1300-*LbMYB48-GFP* over-expression vector was constructed and transformed into *Agrobacterium* GV3101. The subcellular localization experiment was performed using *Agrobacterium* infecting *Nicotiana benthamiana* epidermal cells. The tobacco plants infected by *Agrobacterium* GV3101 were cultured in the dark for 36 h, and the epidermis of the leaves were torn to make patches and stained with 6-diamidino-2-phenylindole (DAPI), and observed by two-photon confocal microscope (Kokkirala et al., 2010; Leng et al., 2021).

### *Cis*-acting element analysis of LbMYB48 promoter sequence

The promoter of *LbMYB48* was specifically amplified by PCR reaction with forward and reverse primers taking the DNA of *L. bicolor* as template (**Supplementary Table 1**). The obtained fragments were sequenced, and the *cis*-acting elements of the promoter were analyzed by Plant CARE software to predict the function of the *LbMYB48* (Han et al., 2019).

### Expression analysis of *LbMYB48*

*L. bicolor* seedlings were cultivated on a normal Murashige and Skoog (MS) medium for 6 d to analyze the expression pattern of *LbMYB48*. Later, the seedlings were transferred to different MS mediums containing 200 mM NaCl, 360 mM mannitol, 5 μM Methyl jasmonate (MeJA), and 1 μM ABA for 0 h, 3 h, 6 h, 12 h, 24 h, and 48 h, respectively. The relative expression levels of *LbMYB48* in different tissue and different treatment conditions were detected by RT-qPCR, and the primers were shown in **Supplementary Table 1** (Wang et al., 2017; Song et al., 2022).

### Transcriptional activation activity of LbMYB48 was assessed using yeast system

A yeast assay system was used to assess whether LbMYB48 has transcriptional activation activity. Amplification of the ORF sequence of the *LbMYB48* was used by homologous recombination primers (**Supplementary Table 1**). The ORF fragment of the *LbMYB48* gene was inserted into the pGBKT7 plasmid and transferred the recombinant plasmid into AH109 yeast (*Saccharomyces cerevisiae*) cells as the experimental group. Empty pGBKT7 plasmid was transferred into AH109 yeast cells as a negative control group, and pGADT7-T and pGBKT7-p53 plasmids were transferred into AH109 yeast cells as a positive control group. Yeast of the experimental, positive, and negative control group was cultured on Synthetic Dropout Media (SD Media). In this assay, 3 SD media (SD/-Trp; SD/-Trp/-His/-Ade and SD/-Trp/-His/-Ade/X-α-Gal) were used to evaluate the transcriptional activation activity of transformed yeast. SD/-TRP is used to confirm the successful transfer of the plasmid into yeast. SD/-Trp/-His/-Ade and X-α-Gal are used to verify the activation of transcription of downstream reporter genes in different groups of yeast. Normal growth and turning blue of yeast is an obvious sign that it has transcriptional activation activity (Maurici et al., 2001; Xu et al., 2016).

### Gene silencing lines obtained by virus induced gene silencing (VIGS)

The 111-588 fragments in the ORF of *LbMYB48* were inserted into the pTRV2 plasmid to construct the pTRV2-*LbMYB48* ^111-588^ plasmid. pTRV2, pTRV2-*LbMYB48* ^111-588^, and auxiliary infection plasmid pTRV1 were transferred into *Agrobacterium* GV3101. The six-leaf wild-type *L. bicolor* was infected by pTRV1, and the pTRV2-*LbMYB48* ^111-588^ mixed bacterial solution was used as the experimental group. The six-leaf wild-type *L. bicolor* infected by pTRV1 and pTRV2 mixed bacterial solution was used as the control group. Dark culture for 2 d after infection (Lu et al., 2020).

### Salt glands density determination in different lines

The leaves of gene-silenced plants and control plants (at the same developmental stage and location) to make standard leaf discs were punched using a hole punch (diameter of 12 mm). The leaf discs were rinsed thoroughly using distilled water to remove the salt on the surface and the number of salt glands per leaf disc was determined by differential-interference microscope observation. Further, 3 leaf discs in the leaves of each silent line were selected to calculate the number of salt glands. The number of salt glands in 3 different parts was counted, and the average of these three parts was considered to be the number of salt glands in one leaf disc for each leaf disc. Later, the average number of 3 leaf discs was the number of salt glands in one transgenic line (Yuan et al., 2018; Lu et al., 2020).

### Determination of salt secretion capacity by leaf disc method

The leaves of gene-silenced plants and the control plants (at the same developmental stage and location) were rinsed using distilled water to remove the salt from the surface, and 10-mm-diameter discs were separated from the leaves. The leaf discs were rapidly dried using filter paper and placed in petri dishes (abaxial surface facing up) containing 30 mL of 200 mM NaCl solution (pH 6.0). The leaf discs were subsequently covered with mineral oil. The secretory droplets above the abaxial surface were collected using a micropipettor after 24 h at 20°C. The volume of secretory droplets per leaf disc (V) was determined, and the concentration of Na^+^ in the fluid (C) was measured using the Dionex ICS-1100 ion chromatography system (Dionex Corp, Sunnyvale, CA, USA). The Na^+^ secretion rate per salt gland was calculated as [V × C/N × time)] (Feng et al., 2014).

### NBT and DAB staining analysis in different lines

ROS can reduce Nitroblue tetrazolium chloride (NBT) to water-insoluble blue formazan and can be employed to indicate the amount of superoxide anion radical ${\text{(O}}_{2}^{-})$ production in plants. Hydrogen peroxide (H_2_O_2_), another ROS in cells, can release oxygen under the action of peroxidase, oxidize diaminobenzidine (DAB), and form a golden yellow precipitate, which can be used to indicate the presence of the hydrogen peroxide content in plant tissues. Hence, the plant tissues were dyed with NBT and DAB staining liquid to infer the degree of salt stress, and the plant tissues were subjected according to the color dark and light (Wang et al., 2007).

### Determination of chlorophyll content in different lines

Different lines of *L. bicolor* were treated with 250 mM NaCl and harvested after 7 d of treatment. Leaves (0.3 g) of each line were put into black test tubes, and 5 mL of dimethyl sulfoxide (DMSO) was added to immerse the leaves in the extract. The tubes were covered with black bags to protect them from light. The samples were heated in the water bath (65°C) until the leaves turned white. The final volume was adjusted to 25 mL with 80% acetone, and the absorbance at 663 nm (A_663_) and 645 nm (A_645_) was measured, with 80% acetone as the blank control. The chlorophyll content was calculated according to the equations (Qiu et al., 2016; Taïbi et al., 2016):


Chl a(mmol/gFW)=(12.7A663−2.69A645)×LFW

Chl b(mmol/gFW)=(22.9A645−4.68A663)×LFW

where L is the constant volume, and FW is the fresh weight (Duan et al., 2018).

### Generation of *LbMYB48* Heterologous Over-expression Lines in Arabidospsis

Generating *LbMYB48* over-expression Arabidopsis is of great significance for verifying the function of this gene. The amplification of the ORF sequence of *LbMYB48* using forward and reverse primers (**Supplementary Table 1**) and the amplified sequence were inserted into the pCAMBIA1300 over-expression vector. Later, the recombinant plasmid was transformed into GV3101 *Agrobacterium*, and age-appropriate wild-type (Col-0) *A. thaliana* was transformed with *Agrobacterium*. Over-expression lines OE 1 and OE 5 in transgenic *A. thaliana* were used for experimental analysis (Zhang et al., 2006).

### Germination percentage assay of different transgenic *A. thaliana* lines

The treated *A. thaliana* individuals whose radicles were able to breach the seed coat within 24 h were considered as successfully germinated seeds. The experiment was repeated three times (Ranal and Santana, 2006; Xu et al., 2021b). The germination percentage (GP, %) was calculated using the following equation.


$$\text{GP=}\frac{number of seeds germinated in 24h}{\text{total number of seeds}}\times 100$$

### Root length measurement of different transgenic *A. thaliana* lines

The root length of different *A. thaliana* lines were measured using ImageJ software. The software measures the relative length of *A. thaliana* roots in the photos and calculates the actual length through proportion. Six replicates were performed in this experiment (Abràmoff et al., 2004; Du et al., 2017).

### Measurement of Na^+^ and K^+^ contents in different lines

Wild type *A. thaliana* and *LbMYB48* over-expression lines were grown for 20 d and treated with half strength Hoagland solution with 150 mM NaCl for 2 weeks. Different lines of *L. bicolor* were treated with 250 mM NaCl and harvested after 7 d of treatment. The contents of sodium and potassium in leaves of different lines at the same leaf position were determined using Flame Photometer (JC-YZ-600, China). Three replicates were set up for this experiment to ensure the accuracy of the results (Munns et al., 2010).

### Measurement of relative electrolytic conductivity in different lines

The *L. bicolor* leaves in the same position under different treatments were sampled and washed thrice with ddH_2_O water. Later, a 0.2 g sample was immersed in distilled water for 0.5 h, placed into test tubes containing 5 mL of ddH_2_O, and incubated at room temperature on a shaker (80 rpm/min) for 3 h (WD-9405B). A conductivity meter (DDS-307 type) was used to measure the conductivity value (r1) of the solution. Prior to the measurement, the samples were boiled in ddH_2_O for 30 minutes and cooled to room temperature, and the conductivity value (r2) of the solution was measured. The REC % was calculated according to the equation (Deans et al., 1995; Tang et al., 2013):


$$REC=\frac{r1}{r2}\times 100$$

### Measurement of malondialdehyde contents in different lines

The content of MDA in the leaves of different lines of *A. thaliana* and *L. bicolor* was measured. The leaves of *A. thaliana* and *L. bicolor* in the same position were mixed with 0.5% thiobarbituric acid (TCA) solution and 0.1% TCA solution in a test tube and boiled for 10 min. The test tubes were immediately placed on ice to cool. Later, the liquid from the test tube was centrifuged at 3000 g for 10 minutes. The MDA content was measured spectrophotometrically by recording the absorbance of the supernatant at 450, 532, and 600 nm. The MDA content was calculated using the following formula:


$$MDA(mM/gFW)=\Delta A\times \left(\frac{V}{155}\right)\times W$$

where △A is the difference between the absorbance of the supernatant at 532 nm and 600 nm; V is the total volume of the supernatant; W represents the fresh weight of the plant material. The absorption coefficient of 1 mM trimethoate at 532 nm is 155. Three replicates were set up for this experiment to ensure the accuracy of the results (Tsikas, 2017).

### Measurements of H_2_O_2_ and O_2_^-^

The content of H_2_O_2_ and ${\text{O}}_{2}^{-}$ in different lines of leaf disc in *L. bicolor* was determined using a UV spectrophotometer (UV756, Shanghai Youke Co., Ltd.). H_2_O_2_ and titanium sulfate (or titanium chloride) form a yellow precipitate of peroxide titanium complex, which was dissolved in sulphuric acid (2M) and determined by colorimetry at 415nm. The ${\text{O}}_{2}^{-}$ concentration was determined in plants by ${\text{O}}_{2}^{-}$ hydroxylamine oxidation. According to the above principle, the standard curve was generated with a standard H_2_O_2_ and ${\text{O}}_{2}^{-}$ concentration gradient solution, and the regression curve between the H_2_O_2_ and ${\text{O}}_{2}^{-}$ concentration and absorption value was obtained (Li et al., 2022).

### Determination of proline contents in different transgenic *A. thaliana* lines

Rosette leaves (0.2 g) were collected from different lines of *A. thaliana* and placed at the bottom of different test tubes. 5 mL of 3% sulfosalicylic acid was added to each test tube and boiled for 10 minutes. The mixture was centrifuged at 3000 g for 3 minutes. Later, 2 mL of the obtained supernatant was collected into fresh tubes, and 2 mL of acetic acid and 3 mL of chromogenic solution were added. The solution in the test tube was boiled for 40 minutes and extracted with 4 mL toluene. The absorbance of the extracted solution was determined at 520 nm wavelength. The formula is as follows:


$$\text{Proline (}\mu \text{g/gFW)=X}\times \left(\frac{V_{t}}{W}\right)\times V_{s}$$

Where X, V_t_, W, and V_s_ refer to the quantified from the standard curve, the volume of extract, the mass of the sample, and the volume of sample, respectively (Ábrahám et al., 2010).

### Determination of soluble sugar contents in different transgenic *A. thaliana* lines

The leaves of the same leaf position of *A. thaliana* with different treatments were taken and rinsed with ddH_2_O. The surface was dried with absorbent paper, cut into pieces, and mixed well. Later, 0.3 g of sample was weighed, and 10 mL of ddH_2_O was added to a test tube with a stopper. The tubes were placed in a boiling water bath for 50 min. The solution in the test tube was filtered and diluted to 25 mL. 0.5 mL of the extract was placed into a 20 mL graduated test tube, and 1.5 mL of distilled water was added followed by the addition of 0.5 mL of anthrone ethyl acetate to the test tube. 5 mL of concentrated sulfuric acid was added to the wall, stirred, and immediately the test tube was placed in a boiling water bath and heated for 1 min. The tubes were allowed to cool to room temperature. The same procedure was employed to add ddH_2_O as a control. UV spectrophotometer was used to measure optical density at 630 nm. The content of soluble sugar in the samples was calculated from the standard curve. Three replicates per treatment (Han et al., 2014).

### RNA-seq analysis

The expression of *LbMYB48* in TRV::LbMYB48 and TRV::0 lines was detected by RT-qPCR, and the lines with high silencing efficiency were selected for the next experiment. RNA was extracted from leaves of the same leaf position and similar size of the silenced lines and the control lines. RNA purity and integrity were checked using NanoDrop2000 and electrophoresis. Qualified RNA samples were reverse transcribed into cDNA libraries, and then sequenced with Novogene High-throughput sequencing. The raw data of the sequencing machine were subjected to quality control to obtain clean data (for data analysis), the sequence set was then compared to the genome of *L. bicolor* to preliminarily annotate the gene function. Gene expression quantification analysis of gene expression levels in the data was performed using the Feature Counts tool in subread software. Quantitation of gene expression was scaled using FPKM (Fragments Per Kilobase of Transcript per Million Fragments Mapped) values, which was the expected number of fragments per kilobase of transcript sequence per million bases sequenced. It considers the influence of sequencing depth and gene length on fragment count and is the most commonly used method for estimating gene expression levels at present. To obtain differentially expressed genes, raw read counts were first normalized, mainly correcting for sequencing depth. Later, the probability of the hypothesis test (*p*-value) was calculated through the statistical model, and conduct Benjamin-Hochberg (BH) procedure was conducted to ensure that the false discovery rate (FDR) is less than 0.05. Finally, the expression of genes in the silenced lines was compared with that in the control lines, and the differentially expressed genes were selected. In addition, we performed the Kyoto Encyclopedia of Genes and Genomes (KEGG) enrichment analysis of all differential genes for each differential comparison set using cluster Profiler software (Hrdlickova et al., 2017). Some RNA-seq differentially expressed genes were screened and verified by RT-qPCR. The forward and reverse primers of the differentially expressed genes are listed in **Supplementary Table 1**.

### RT-qPCR of stress-related genes

*A. thaliana* seedlings grown on 100mM NaCl MS medium for 7 d were employed as the sample. RT-qPCR was used to detect the difference in the expression of downstream marker genes such as *RD29A*, *RD22*, *AtP5CS1*, and *SOS1* between different *A. thaliana* lines. The forward and reverse primers of these marker genes were shown in **Supplementary Table 1** (Taji et al., 2004; Tuteja, 2007).

### Data analysis and statistics

In this study, SPSS software (version 19.0) was used to statistically analyze the data obtained from the experiment. Three or six replicates were set up to calculate the standard deviation (SD) and ensure accuracy. Different letters (a–g) were used to indicate significant differences between different columns at P< 0.05 (Mccormick and Salcedo, 2017).

## Results

### LbMYB48 is an R1-type MYB transcription factor localized in the nucleus

**Figure 1:**
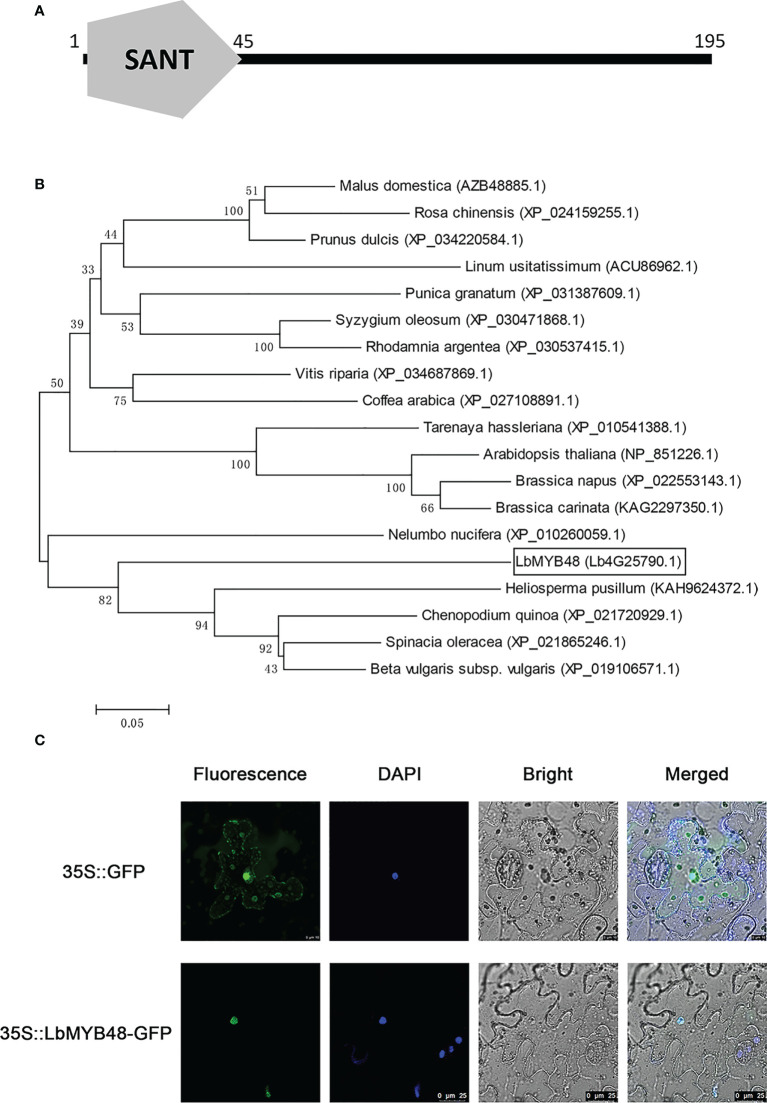
LbMYB48 is an R1-type MYB transcription factor located in the nucleus. **(A)** The conserved domain of LbMYB48 protein; **(B)** Phylogenetic tree analysis of MYB48 related proteins in different species; **(C)** The subcellular localization of LbMYB48 in tobacco.

The open reading frame of the *LbMYB48* gene contains 588 nucleotides and encodes a protein with 195 amino acids. **Figure 1A** shows the presence of only one conserved SANT domain between 1-45 amino acids, suggesting that *LbMYB48* was a typical R1-type MYB transcription factor. The phylogenetic tree displayed that the LbMYB48 is highly homologous to MYB48-like proteins from *Heliosperma pusillum*, *Chenopodium quinoa*, *Spinach oleracea*, and *Beta vulgaris*, which have the characteristics of salt and drought tolerance. It is worth mentioning that, LbMYB48 in *L. bicolor* and AtMYB48 in *A. thaliana* are far homologous (**Figure 1B**). Tobacco subcellular localization experiment demonstrated that the GFP-LbMYB48 fusion expression vector only produced a GFP signal in the nucleus of tobacco epidermal cells (**Figure 1C**), suggesting that LbMYB48 is a typical R1-type MYB transcription factor localized in the nucleus.

### *LbMYB48* was induced by NaCl, drought, MeJA, and ABA

**Figure 2:**
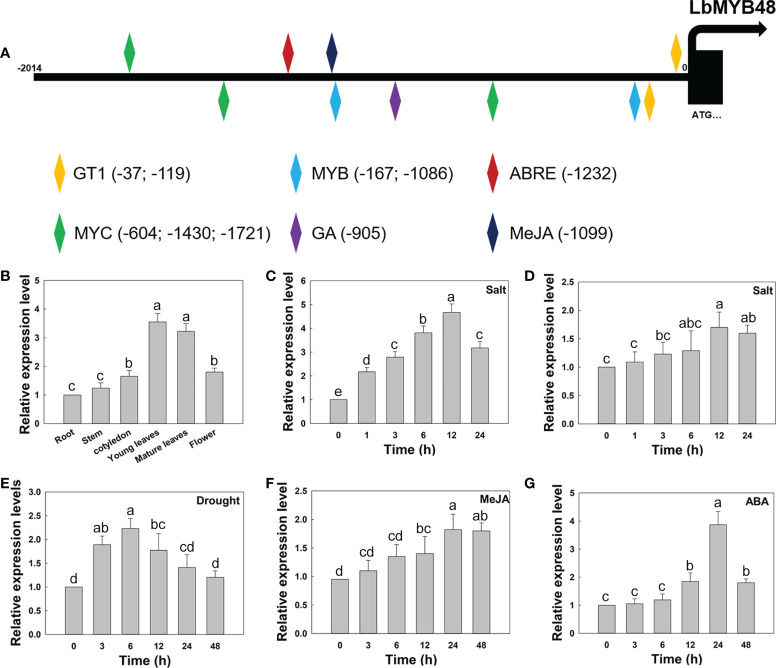
*LbMYB48* is regulated by NaCl, drought and phytohormones. **(A)** Important *cis*-acting elements in the promoter of *LbMYB48*; **(B)** Relative expression levels of *LbMYB48* in different tissues of *L. bicolor*; **(C)** Relative expression levels of *LbMYB48* in the leaves of *L. bicolor* under 250 mM NaCl treatment for different time (1-24 h); **(D)** Relative expression levels of *LbMYB48* in root of *L. bicolor* under 200 mM NaCl treatment (1-24 h); **(E)** Relative expression levels of *LbMYB48* in *L. bicolor* under 360 mM mannitol treatment (3-48 h); **(F)** Relative expression levels of *LbMYB48* in *L. bicolor* under 5 μM MeJA treatment for different time (3-48 h); **(G)** Relative expression levels of *LbMYB48* in *L. bicolor* under 1 μM ABA treatment for different time (3-48 h). Three replicates should be set up to calculate the standard deviation (SD) and ensure accuracy. Different letters (a–e) were used to indicate significant differences between different columns at *P *< 0.05.

The expression pattern of *LbMYB48* and the promoter sequence of *LbMYB48* were cloned and analyzed with Plant CARE software for further clarification. Several *cis*-acting elements related to plant growth, development, and stress, including GT1-motif, ABRE, MRE, LTR, and CGTCA-motif, were contained in the promoter region of *LbMYB48* (**Figure 2A**), suggesting that the expression of *LbMYB48* may be induced by plant hormones and abiotic stresses such as gibberellins (GAs), ABA, MeJA, high salt, and drought. The expression patterns of different tissue sites displayed that *LbMYB48* was mainly expressed in the young and mature leaves of *L. bicolor* (**Figure 2B**). Furthermore, wild-type *L. bicolor* was grown on MS medium for 5 d and subjected to NaCl, drought, MeJA, and ABA treatments to further examine its expression pattern. The experimental results indicated that the expression of *LbMYB48* was detected in different tissues, with the highest expression observed in young leaves. Different treatments found that the expression of *LbMYB48* in the leaves of *L. bicolor* was strongly induced by NaCl. **Figure 2C** shows that the expression levels reached their peak when treated with NaCl for 12 h, which was more than 4.7 times higher than the control. Moreover, the expression of *LbMYB48* in the root was also induced by NaCl, and their expression levels reached the peak at 12 h, which was 1.5 times higher than the control (**Figure 2D**). In addition, the expression of *LbMYB48* increased about 2.3 times when subjected to drought stress for 6 h (**Figure 2E**), about 1.7 times when treated with MeJA for 24 h (**Figure 2F**), and 4 times after being treated with ABA for 24 h (**Figure 2G**), in the whole plant, indicating that *LbMYB48* may be involved in the development process and a variety of abiotic stress responses.

Most MYB transcription factors have transcription factor activity. Therefore, a yeast system was used to analyze the transcriptional activation of LbMYB48. The pGBKT7-*LbMYB48*, pGBKT7-p53+pGADT7-T (positive control), and pGBKT7 (negative control) vectors were introduced into yeast strain AH109. It was observed that all yeast cells could grow on SDO (SD/-TRP) medium. However, yeast cells containing the pGBKT7-*LbMYB48* vector or positive control vectors were able to grow and produced the blue substrate in TDO/X (SD/-Trp/-His/-Ade/X-α-Gal) medium, whereas cells harboring negative control were unable to grow (**Supplementary Figure 1**). Therefore, LbMYB48 has transcriptional activation activity in yeast cells.

### *LbMYB48* positively regulates salt gland development in *L. bicolor*

**Figure 3:**
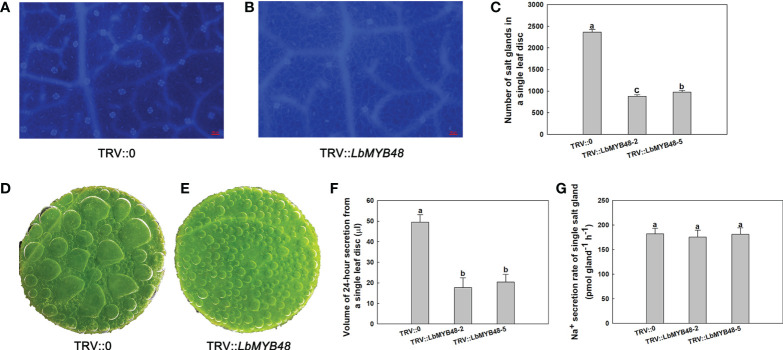
Salt gland detection and leaf disc salt secretion of *LbMYB48*-silenced *L. Bicolor.* **(A)** Distribution of salt glands in TRV*::*0 leaf for control group, the scale bar = 50μm; **(B)** Distribution of salt glands in TRV*::LbMYB48* leaf for experimental group, the scale bar = 50μm; **(C)** The number of salt glands in TRV*::*0 leaf disc for control group and TRV*::LbMYB48* leaf disc for experimental group; **(D)** Salt secretion of control group TRV*::*0; **(E)** Salt secretion of experimental group TRV*::LbMYB48*; **(F)** 24-h secretion of leaf disc of TRV*::*0 for control group and TRV::*LbMYB48* for experimental group; **(G)** Salt secretion per h by individual salt gland of TRV*::*0 and TRV::*LbMYB48*. Three replicates should be set up to calculate the standard deviation (SD) and ensure accuracy. Different letters (a–b) were used to indicate significant differences between different columns at *P* < 0.05.

Gene silencing (TRV-VIGS) system has been established in *L. bicolor*, so VIGS was used to investigate the function of *LbMYB48* in *L. bicolor*. Gene silencing was performed by using wild-type *L. bicolor* at the six-leaf stage. Empty plasmid TRV::0 as control and TRV::*LbMYB48* plasmid as the experimental group was transformed into the leaves of *L. bicolor*, respectively. The detection of relative expression levels showed that VIGS had high silencing efficiency in TRV::*LbMYB48* lines (Supplementary Figure 2). To investigate whether *LbMYB48* gene silencing affects salt gland development, the density of salt glands was observed and counted by microscope (**Figures 3A, B**). The results showed that the silencing of *LbMYB48* resulted in a significant decrease in the density of salt glands, which was about 30% of that of the control group (**Figure 3C**). Scanning electron microscopy (SEM) showed that there were no significant differences in the area of other epidermal cells among the different lines (**Supplementary Figure 3**). This further indicated that the reduction of salt glands in the silent line was not caused by the increased area of other epidermal cells, but by the decreased differentiation of salt glands. Further, to investigate whether *LbMYB48* gene silencing affects salt secretion ability, the leaf disc of different liens was placed on the surface of 200 mM NaCl solution, and the side of the leaf disc in contact with air was sealed with mineral oil. After 24 h, the secreted fluid of the leaf disc of the *LbMYB48* silencing lines decreased significantly compared with the control (**Figures 3D, E**). The secretion of a single leaf disc was carefully collected and secreted fluid volume was measured. The experimental results found that the total volume of salt secreted by individual leaf discs was significantly reduced in silent lines (**Figure 3F**), and the secretion rate of a single salt gland was not significantly different between the wild-type and *LbMYB48*-silenced plants (**Figure 3G**). Therefore, it indicates that the main reason for the significant decrease in the salt secretion of the *LbMYB48* silenced line is the significant decrease in the number in salt glands rather than the structure of the salt glands.

### LbMYB48 positively regulates the salt-tolerance ability of *L. bicolor*

**Figure 4:**
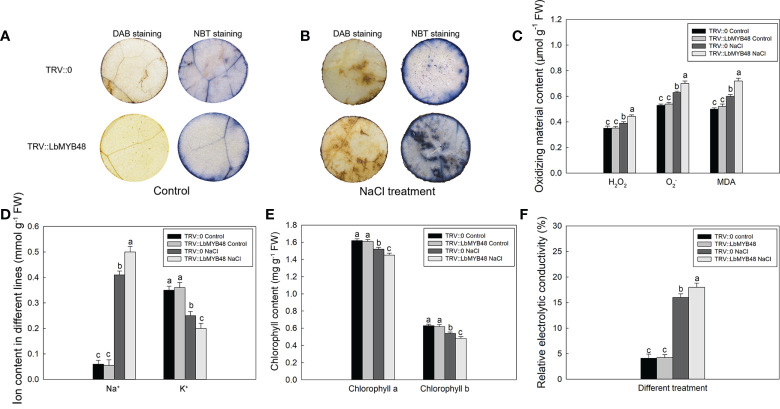
Analysis of salt tolerance capacity of *LbMYB48* silenced lines. **(A)** DAB and NBT staining results of leaf discs from the TRV*:: LbMYB48* and TRV:: 0 lines under normal growth conditions; **(B)** DAB and NBT staining results of leaf discs from the TRV*:: LbMYB48* and TRV:: 0 lines under NaCl treatment; **(C)** the H_2_O_2_, ${\text{O}}_{2}^{-}$ and MDA content in TRV*:: LbMYB48* and TRV:: 0 lines; **(D)** Na^+^ and K^+^ contents in TRV*::LbMYB48* and TRV:: 0 lines; **(E)** Chlorophyll a and chlorophyll b contents in TRV*:: LbMYB48* and TRV:: 0 lines; **(F)** Relative electrolytic conductivity analysis of TRV*:: LbMYB48* and TRV:: 0 lines. Three replicates should be set up to calculate the standard deviation (SD) and ensure accuracy. Different letters (a–c) were used to indicate significant differences between different columns at *P* < 0.05.

*L. bicolor* is a recretohalophyte, and the main factor that determines its salt tolerance is the salt secretion ability of the salt gland. Moreover, the development of salt glands is closely related to their salt secretion capacity. The decrease of *LbMYB48* gene expression led to a sharp decrease in the density of salt glands, so we speculated that the salt tolerance of the *LbMYB48-*silenced lines was deteriorated. The different lines of TRV::*LbMYB48* and TRV::0 were treated in 250 mM NaCl solution for 7 d, and then the physiological parameters of salt tolerance such as NBT and DAB staining, endogenous ROS (H_2_O_2_ and ${\text{O}}_{2}^{-}$ ), malondialdehyde (MDA), ion content, chlorophyll content and relative electrolytic conductivity (REC) were determined. The results demonstrated that the degree of NBT staining and DAB staining of TRV*::LbMYB48* was deeper than that of TRV*::*0 under salt treatment conditions (**Figures 4A, B**). Furthermore, the deeper NBT staining, the more superoxide radicals ${\text{(O}}_{2}^{-})$ are contained in plants, the deeper DAB staining, the higher H_2_O_2_ content in leaves, correspondingly, there was more MDA content in the TRV::*LbMYB48* silencing lines (**Figure 4C**). In addition, more Na^+^ (**Figure 4D**), less chlorophyll content (**Figure 4E**), and higher REC (**Figure 4F**) were contained in the *LbMYB48* gene-silencing lines. Therefore, the salt tolerance of *LbMYB48-*silenced lines was reduced with a decrease in the salt glands per unit leaf area. This indicates that LbMYB48, as a transcription factor, plays an important role in the regulation of salt gland development and plant salt tolerance.

### RNA-sequencing of the control and *LbMYB48* silenced *L. bicolor*

**Figure 5:**
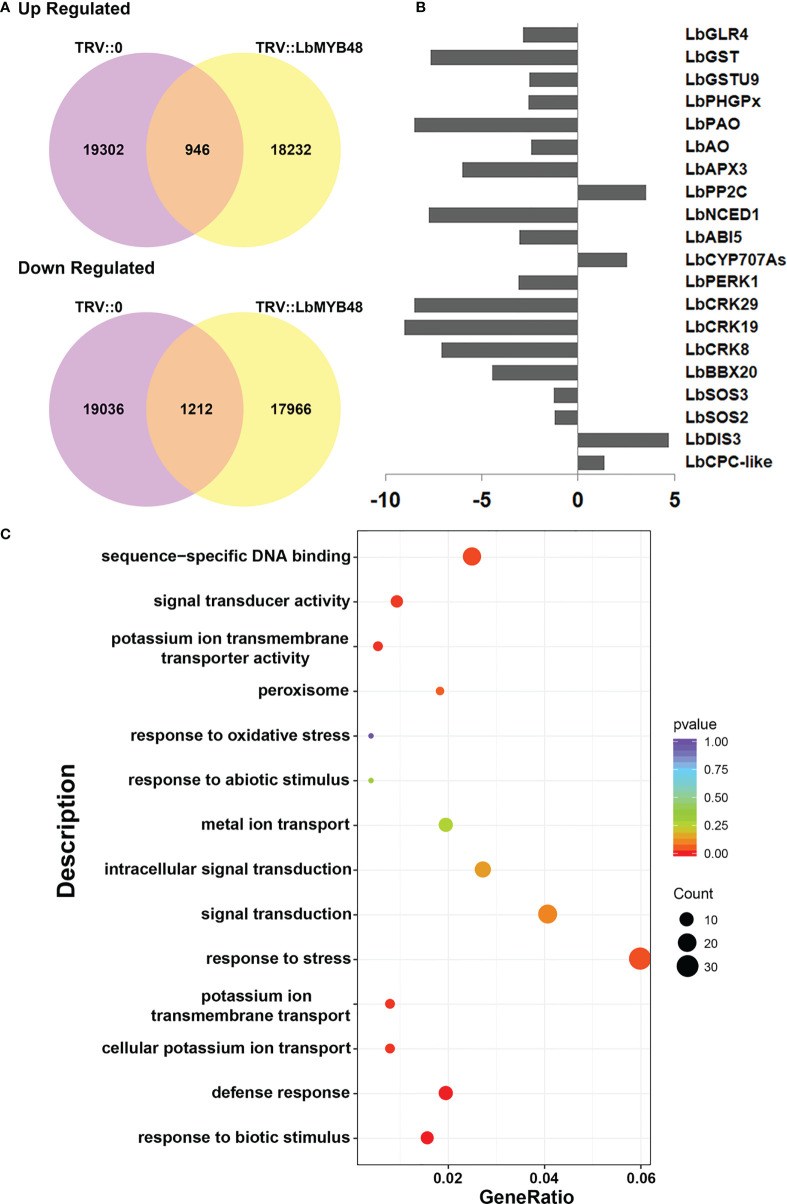
Analysis of RNA-seq of *LbMYB48*-silenced *L. bicolor* lines. **(A)** Venn diagram of up-regulated differentially expressed genes and down-regulated differentially expressed genes in TRV:: *LbMYB48* and TRV::0 lines; **(B)** Differentially expressed genes found in RNA-seq experiments related to the development of plant epidermal cells (salt glands), salt stress, ABA signaling pathway and ROS scavenging; **(C)** KEGG signal pathway enrichment analysis of differentially expressed genes in *LbMYB48* silenced lines.

The salt gland density and the ability of *L. bicolor* to secrete salt were significantly reduced after *LbMYB48* silencing. The possible molecular mechanisms were analyzed by RNA-seq. RNA from leaves with the same leaf position and same growth trend of TRV::*LbMYB48* and TRV::0 was extracted and the RNA-seq was performed to search for differentially expressed genes that might contribute to this phenotype. **Figure 5A** displays the statistical alignment of FPKM values of all genes in the results of RNA-seq High-throughput sequencing. 2158 differentially expressed genes were filtered out, 1212 genes were down-regulated and 946 genes were up-regulated in the *LbMYB48* silenced lines compared with the control lines (**Figure 5A**). Additionally, RNA-seq experiments were employed to screen some differentially expressed genes that might be related to the decreased salt gland density and salt tolerance of the silenced lines. Specifically, *LbCPC-like* and *LbDIS3*, which are related to plant epidermal cell (salt gland) development; *LbSOS2*, *LbSOS3*, *LbBBX20*, and *LbCRKs*, which are related to plant salt tolerance; *LbCYP707As*, *LbABI5*, *LbNCED1*, and *LbPP2C*, that are related to ABA signaling pathway; *LbAPX3*, *LbPAO*, *LbPHGPx*, *LbGSTs*, and *LbGLR4* that are related to plant ROS scavenging (**Figure 5B**). In order to confirm the accuracy of the RNA-seq results, some differentially expressed genes in this experiment were selected and validated by RT-qPCR. The RT-qPCR and RNA-seq results were in agreement, indicating that the experimental result of this RNA-seq were reliable (**Supplementary Figure 4**). Additionally, the KEGG analysis of differentially expressed genes in RNA-seq was also performed, which suggested that the genes related to “response to stress” accounted for the highest proportion (**Figure 5C**). However, further research is necessary to comprehend how *LbMYB48* regulates these genes and their functions in salt tolerance.

### *LbMYB48* heterologous over-expression improves salt tolerance of transgenic *A. thaliana*

**Figure 6:**
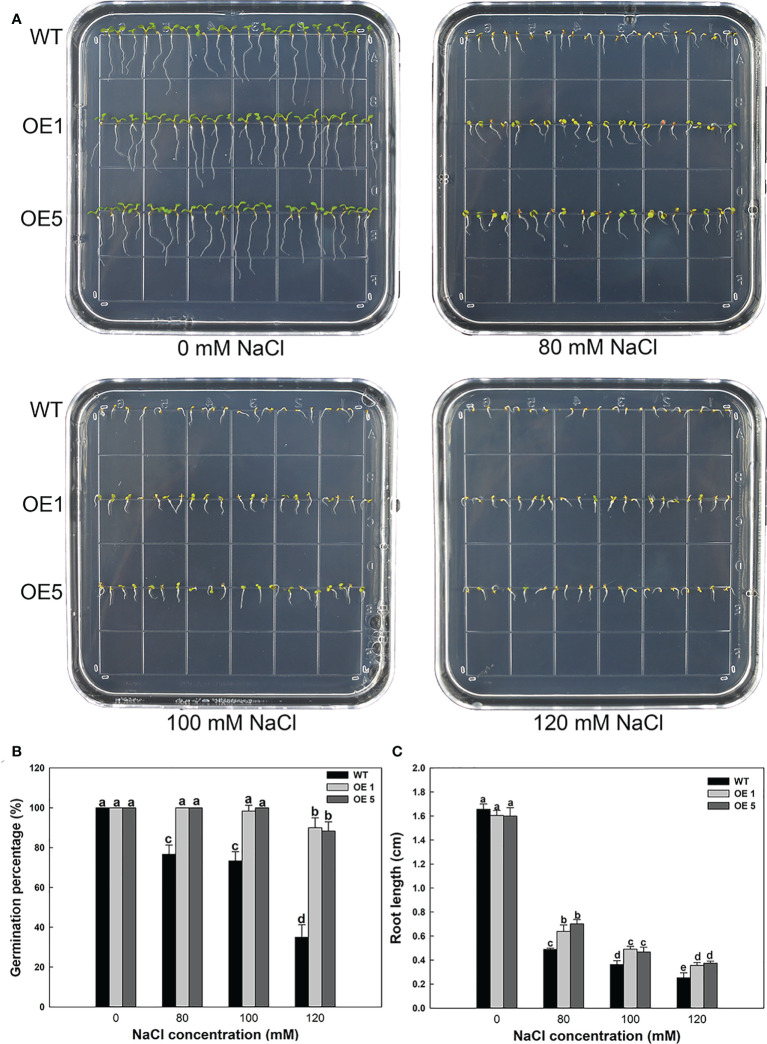
Heterologous expression of *LbMYB48* enhances salt tolerance at the seedling stage in transgenic *A. thaliana*. **(A)** Phenotype of *LbMYB48* over-expression lines versus wild-type lines grown for 5 d under different NaCl concentrations; **(B)** 24 h germination percentage of the wild-type and two over-expression lines treated with different concentrations of NaCl; **(C)** Root length of wild-type *A. thaliana* and two over-expression lines grown for 5 d under different salt concentrations. This experiment had three replicates to calculate the standard deviation (SD). Different letters (a–g) were used to indicate significant differences between different columns at *P* < 0.05.

LbMYB48 has higher homology with some salt-tolerant plants, hence it is of great significance to study the role of this gene in non-halophyte plants in salt tolerance, which is crucial to improve the salt resistance of crops. *A. thaliana* wild-type seeds of and homozygous seeds of transgenic *LbMYB48* were seeded into the MS medium containing different concentrations of NaCl, and seed germination and the growth status of the seedlings were observed daily. The phenotypes were photographed and recorded on the fifth day, and the relevant physiological indexes of wild-type and each transgenic line under different degrees of salt stress were statistically analyzed. **Figure 6A** shows that on the MS medium without NaCl, the growth of wild-type *A. thaliana* and over-expression *A. thaliana* are the same. However, on the 80, 100 and 120 mM NaCl MS medium, the growth of *LbMYB48* over-expression *A. thaliana* is significantly better than that of wild-type *A. thaliana*. Moreover, compared with normal conditions, *A. thaliana* grows on 120 mM NaCl MS medium, and the germination percentage of wild-type and two over-expression lines decreased by 65%, 10%, and 11.7%, respectively (**Figure 6B**). The root lengths of wild-type and over-expression lines were reduced by 84.8%, 78.1%, and 76.8%, respectively, compared to the controls (**Figure 6C**).

**Figure 7:**
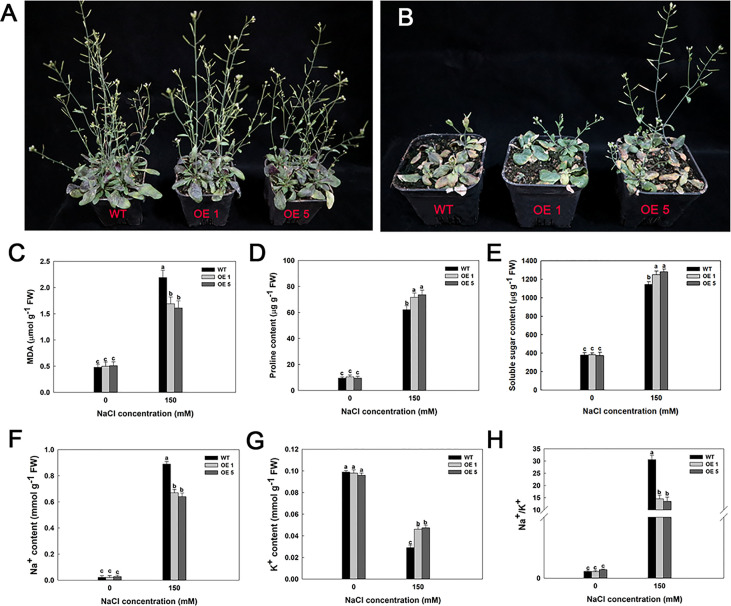
Phenotypes and physiological indexes of LbMYB48 over-expression lines and wild-type lines at maturity stage under 0 and 150 mM NaCl. **(A)** Different genotypes under normal growth condition; **(B)** Different genotypes under 150 mM NaCl for 14 d; **(C)** MDA content; **(D)** Proline content; **(E)** Soluble sugar content; **(F)** Na+ content; **(G)** K+ content; **(H)** Ratio of Na+ content to K+ content. Three replicates were used to calculate the standard deviation (SD) and ensure accuracy. Different letters (a–c) were used to indicate significant differences between different columns at P < 0.05.

Furthermore, several *LbMYB48* over-expression lines and wild-type *A. thaliana* were planted in small flowerpots to determine whether the *LbMYB48* over-expression lines have improved salt tolerance compared to the wild type at maturity. After 14 days of growth, the plants with the same growth trend were taken for salt treatment. The treatment method involved pouring of 50 ml 1/2 Hoagland nutrient solution with 150 mM NaCl concentration into each small flowerpot every day. Simultaneously, the treatment method of the control group involved pouring of 50 ml 1/2 Hoagland nutrient solution without salt into each small flowerpot every day. The phenotype was observed after 14 days of continuous treatment, which indicated that under high salt stress, the growth of *LbMYB48* over-expression lines was still better than that of wild-type *A. thaliana* (**Figures 7A, B**). The MDA content and proline content of the *LbMYB48* over-expression lines and wild-type *A. thaliana* was measured. It was found that the MDA content in the wild-type *A. thaliana* is much greater than that of the *LbMYB48* over-expression lines (**Figure 7C**), indicating that the damage of salt stress to the wild-type *A. thaliana* is much severe than that of the *LbMYB48* over-expression lines. In addition, the proline content in the *LbMYB48* over-expression lines was much higher than that in the wild-type *A. thaliana* (**Figure 7D**) indicating that *LbMYB48* might positively regulate salt tolerance in transgenic *A. thaliana* by affecting proline synthesis. **Figure 7E** displays that similar to proline, soluble sugars have the same trend in different lines.

In addition, the Na^+^ content, K^+^ content, and Na^+^/K^+^ ratio in leaves of the *LbMYB48* over-expression lines versus wild-type *A. thaliana* under normal culture and high salt treatment were measured. It was observed that under normal culture conditions, there is no obvious difference in Na^+^ content, K^+^ content, and Na^+^/K^+^ ratio between the leaves of *LbMYB48* over-expression lines and wild-type *A. thaliana*. However, the Na^+^ content in the leaves of *LbMYB48* over-expression lines was much lower than that of wild-type *A. thaliana* under the treatment of 150 mM NaCl (**Figure 7F**). The K^+^ content in the leaves of *LbMYB48* over-expression lines was higher than that of wild-type *A. thaliana* (**Figure 7G**), and the Na^+^/K^+^ ratio of *LbMYB48* over-expression lines and significantly lower than that of wild-type *A. thaliana* (**Figure 7H**). These results indicated that under salt stress, the *LbMYB48* over-expression line was subjected to much less stress than the wild-type *A. thaliana*, and *LbMYB48* may positively regulate salt tolerance in transgenic *A. thaliana* by enhancing ion homeostasis.

**Figure 8:**
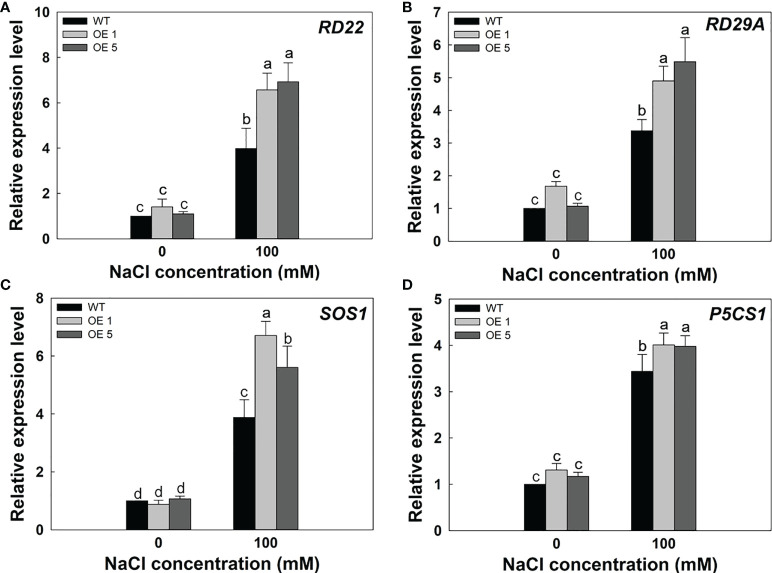
Expression levels of salt tolerance marker genes in LbMYB48 over-expression lines and wild-type A. thaliana. **(A)** Relative expression level of RD22; **(B)** Relative expression level of RD29A; **(C)** Relative expression level of SOS1; **(D)** Relative expression level of P5CS1. Three replicates were used to calculate the standard deviation (SD) and ensure accuracy. Different letters (a–d) were used to indicate significant differences between different columns at P<0.05.

To further verify the molecular mechanism of *LbMYB48* enhancing salt tolerance in *A. thaliana*. We performed RT-qPCR experiments on *A. thaliana* seedlings of various lines treated with 100 mM NaCl to detect the expression level of marker genes related to salt stress. Under salt treatment, *RD22*, *RD29A*, *SOS1*, and *P5CS1* were more strongly induced in *LbMYB48* over-expression plants than in the wild-type. *RD22* and *RD29A* both participate in the ABA signaling pathway, *SOS1* is an ion transporter marker gene, and *P5CS1* is a proline synthesis marker gene (**Figure 8**). These data indicate that *LbMYB48* is likely to improve salt tolerance in *A. thaliana* through the ABA signaling pathway and the proline synthesis signaling pathway.

## Discussion

*L. bicolor* is known as the “pioneer plant” for the transformation of saline-alkali land (Xu et al., 2021a). The salt gland structure of *L. bicolor* and its strong salt tolerance enable it to grow normally and complete its life cycle in a saline environment (Leng et al., 2019). At present, there have been several studies on the development of salt glands, salt secretion mechanism, and resistance-related genes of *L. bicolor*, which have received tremendous attention (Feng et al., 2015). Especially with the annotation of the *L. bicolor* genome (Yuan et al., 2022), further excavation and elucidation of the molecular mechanism of the key genes in the development of the salt gland structure and the salt secretion mechanism are the key content to further reveal the salt tolerance characteristics of halophytes (Leng et al., 2018).

Several previous studies have found that genes expressed early in salt gland development and salt-induced may play critical roles in salt gland development and salt tolerance of *L. bicolor* (Yuan et al., 2022). *LbMYB48* was identified as an R1-MYB transcription factor in *L. bicolor* (**Figure 1B**), which is expressed in the early stage of salt gland development and was significantly induced by salt stress. MYB48 family proteins in different plants have been studied to some extent, the R1-MYB transcription factor ZmMYB48 was isolated from maize (Zea mays L.) (Wang et al., 2017). Quantitative RT-PCR analysis indicated that the expression of the *ZmMYB48* gene was induced by drought and ABA treatment. Subcellular localization analysis showed that ZmMYB48 protein localized the nucleus of tobacco leaf epidermal cells. Transactivation assays in yeast demonstrate that ZmMYB48 has transcriptional activation capacity. Heterologous over-expression of *ZmMYB48* in *A. thaliana* significantly increased plant tolerance to drought stress, as determined by physiological analysis of survival rate, relative water content, MDA content, REC, and proline content. Furthermore, over-expression of *ZmMYB48* enhanced the expression of stress ABA-responsive genes such as *P5CS1*, *RD22*, *RD29B*, and *ABI1*. Furthermore, under drought stress, *ZmMYB48*-overexpressing plants accumulated higher levels of ABA than wild-type plants. These results suggest that ZmMYB48 may be a positive regulator of drought stress response through ABA signaling. Another study showed that the *OsMYB48-1* gene in rice (Xiong et al., 2014) was strongly induced by polyethylene glycol (PEG), ABA, H_2_O_2_, and dehydration, while high salinity and cold treatments slightly induces the expression of *OsMYB48-1*. The OsMYB48-1 protein is localized in the nucleus and has transactivation activity at the C-terminus. Over-expression of *OsMYB48-1* in rice significantly increased tolerance to simulated drought and salt stress induced by mannitol, PEG, and NaCl. Compared with wild-type plants, over-expression lines exhibited reduced water loss, lower MDA content, and higher proline content under stress conditions. In addition, overexpressing plants were more sensitive to ABA at the germination and post-emergence stages and accumulated more endogenous ABA under drought stress conditions. Further studies demonstrated that over-expression of *OsMYB48-1* could regulate the expression of some ABA biosynthesis genes (*OsNCED4* and *OsNCED5*), early signal genes (*OsPP2C68* and *OSRK1*), and late response genes (*RAB21*, *OsLEA3*, *RAB16C*, and *RAB16D*) under drought stress conditions. These results suggest that OsMYB48-1 functions as a novel MYB-related transcription factor that positively regulates drought and salt tolerance by regulating stress-induced ABA synthesis. Moreover, AtMYB48 in *A. thaliana* has not been studied, but due to the high homology between AtMYB48 and AtMYB59, it is speculated that AtMYB48 has similar functions. The *A. thaliana* transcription factor AtMYB59 regulates K^+^/NO3^-^ transport under low potassium conditions. The *A. thaliana* transcription factor AtMYB59 is a positive transcriptional regulator of the *A. thaliana* nitrate transporter NRT1.5 (Mu et al., 2009). Although LbMYB48 shares some homology with these non-halophytes such as maize and rice, LbMYB48 showed more high amino acid sequence similarity and close homology to MYB48 like proteins from the halophytes *Chenopodium quinoa*, whereas it shows low similarity and distant homology to AtMYB48 protein from *A. thaliana* (**Figure 1B**), it can be seen from this, the function of LbMYB48 should have a large difference with MYB48 like proteins in non-halophyte. Therefore, studying the *LbMYB48* gene can lead to a more comprehensive understanding of the role of salt gland development and salt tolerance in plants.

The subcellular localization experiment of *LbMYB48* demonstrated that this protein was localized in the nucleus (**Figure 1C**), and the transcriptional activation activity experiment exhibited that this protein has transcriptional activation activity (**Supplementary Figure 1**). Nuclear localized proteins often act like transcription factors, and transcriptional activation activity assay can predict that *LbMYB48* plays a regulatory role by activating the expression of downstream target genes (Garcia-Bustos et al., 1991). In addition, the *cis*-acting elements in response to high salt, drought, and other stresses, ABA, MeJA, and other hormones in the promoter of *LbMYB48*, especially GT1, which is a specific *cis*-acting element in response to salt were identified (Xu et al., 2018). Correspondingly, expression pattern analysis also further proved that LbMYB48 was rapidly expressed in response to salt stress and stress hormones. These results suggested that LbMYB48 may function as a transcriptional activator to regulate the transcription of downstream target genes to participate in the development of salt glands of *L. bicolor* and abiotic stress tolerance.

The density of salt glands (**Figure 3**) and the ability of salt tolerance (**Figure 4**) were significantly decreased in the *LbMYB48* silent lines. Moreover, studies have shown that transcription factors are involved in the differentiation of plant epidermal structure and often play a key regulatory role upstream of the epidermal structure development signaling pathway. For example, in the development of trichome and root hairs of model plants such as *A. thaliana*, tomato, and *Artemisia annua*, MYB-type transcription factors such as GL1, TTG1, CPC, and AaMIXTA1 play a key role in the fate-determining stage of trichome and root hair development (Han et al., 2022). Unlike *A. thaliana* and other non-halophyte, the leaves of *L. bicolor* have no trichome structure, but the time node of the initial development of *L. bicolor* salt glands is almost the same as that of *A. thaliana* trichome, which is earlier than the stomata. There is no salt gland adjacent phenomenon in *L. bicolor*, and there is no trichome adjacent phenomenon in *A. thaliana* (Han et al., 2021). Therefore, we speculate that the development of salt glands and trichomes may have a common origin, and the key genes of trichome development may also regulate the development of salt glands (Yuan et al., 2019). *LbCPC-like* and *AtCPC* are homologous genes, and AtCPC can form complexes with TTG1 and GL3 to negatively regulate the development of trichome. *AtCPC* over-expression *A. thaliana* plants have fewer trichome than wild-type plants (Wada et al., 1997; Tominaga et al., 2007). Furthermore, according to our experimental results, the number of salt glands in the silenced lines was significantly lower than that in the control lines, and the expression of *LbCPC-like* was up-regulated in the silenced lines, due to *LbMYB48* was a transcriptional activator, indicating that *LbMYB48* may indirectly negatively regulate the expression of *LbCPC-like*, thereby regulating the development of salt glands in *L. bicolor*, and then affect its salt tolerance. In addition, the *A. thaliana DIS3* is critical for trichome morphogenesis, and *DIS3* knockout *A. thaliana* mutants exhibit distorted trichome deformation, cell-to-cell adhesion defects, and reduced hypocotyl growth in etiolated seedlings (Basu et al., 2005). The *LbDIS3* gene was also up-regulated in the *LbMYB48* silent line, therefore, *LbDIS3* may also negatively regulate the development of salt glands under the regulation of LbMYB48 in an indirect way.

Furthermore, LbMYB48 may also directly or indirectly regulate the salt tolerance of *L. bicolor* by regulating other differentially expressed genes such as ion transport-related genes *LbSOS2*/*LbSOS3* (Qiu et al., 2002), ABA signaling-pathway related genes *LbABI5* (Skubacz et al., 2016), salt stress-related genes *LbRLKs* (Ye et al., 2017) and ROS scavenging related genes *LbGSTs* (Yang et al., 2014). For *LbMYB48* transgenic *A. thaliana*, physiological indicators and molecular tests showed a similar salt-tolerance mechanism to *L. bicolor*. In the past few years, there are several genes from *L. bicolor* that significantly enhance the salt tolerance of the non-halophyte *A. thaliana*. For instance, *LbSAD2* from *L. bicolor* is a homolog of *SAD2* in *A. thaliana*, which enhances salt tolerance of transgenic *A. thaliana* by specifically reducing Na^+^ accumulation and ABA sensitivity (Zhang et al., 2021). MYB transcription factor LbTRY from halophyte *L. bicolor* was found to reduce the salt tolerance of transgenic *A. thaliana* by decreasing *SOS* gene expression and proline accumulation (Leng et al., 2021). In addition, according to the latest study, the *Lb1G04899* gene in *L. bicolor* could tolerate the salt stress of transgenic *A. thaliana* by increasing osmotic tolerance (Wang et al., 2021b). But the specific salt resistance molecular mechanism needs to be further studied.

**Figure 9:**
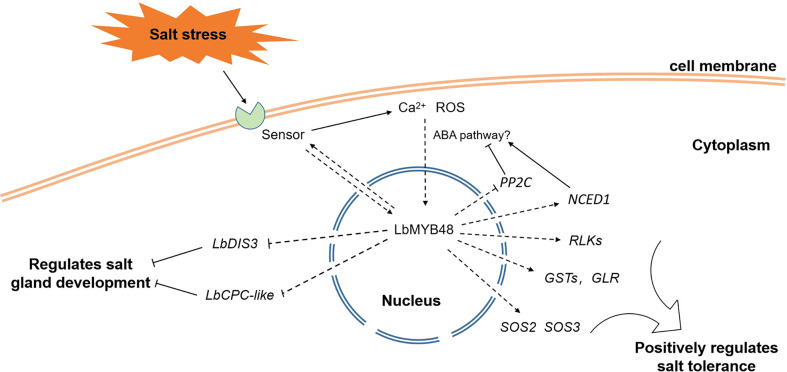
A possible model for *LbMYB48* regulation of salt gland development and plant salt tolerance.

**Figure 9** illustrates the proposed model for the regulation of salt gland development and plant salt tolerance by LbMYB48 through a series of experiments. Salt stress-induced gene *LbMYB48* may act as a transcription factor and indirectly regulate the expression of *LbCPC-like* and *LbDIS3* to positively regulate the development of salt glands, and then positively regulate the salt secretion ability of *L. bicolor*. At the same time, salt stress experiments in *L. bicolor* and transgenic *A. thaliana* also showed that *LbMYB48* has the function of positively regulating salt tolerance of plants by affecting the expression of salt tolerance-related genes such as *LbSOS2*, *LbSOS3*, *LbGSTs*, and *LbRLKs*. In addition, the heterologous expression of *LbMYB48* in *A. thaliana* further proved that the positive regulatory role of this gene in plant salt tolerance is consistent, and it is likely to indirectly participate in the ABA signaling pathway.

Salt secretion from salt glands is the most critical pathway in the salt-tolerant strategy of *L. bicolor*. Although several studies have made a relatively systematic elucidation of the mechanism of salt gland development and salt secretion, the possible mechanism of salt gland development and secretion has been speculated. But there are still many critical issues to be resolved. The in-depth study of gene regulation levels can better explain the salt gland development and salt resistance mechanism of *L. bicolor*, and the mining of related salt tolerance genes is of great significance for transgenic engineering to improve crop salt tolerance.

## Data availability statement

The data presented in the study are deposited in the NCBI repository, accession number PRJNA890322.

## Author contributions

GH and ZQ wrote this manuscript. ZQ and YL participated in the experiment. ZY, ZZ, YZ, JG, LL and CW participated in the writing and modification of this manuscript. BW and GH conceptualized the idea. All authors contributed to the article and approved the submitted version.

## Funding

This work was supported by National Natural Science Research Foundation of China (project No. 32000209 and 32170301), Natural Science Research Foundation of Shandong Province (project No. ZR2020QC031) and China Postdoctoral Science Foundation (project No. 2020M672114).

## Conflict of interest

The authors declare that the research was conducted in the absence of any commercial or financial relationships that could be construed as a potential conflict of interest.

## Publisher’s note

All claims expressed in this article are solely those of the authors and do not necessarily represent those of their affiliated organizations, or those of the publisher, the editors and the reviewers. Any product that may be evaluated in this article, or claim that may be made by its manufacturer, is not guaranteed or endorsed by the publisher.

## Supplementary material

Supplementary Figure 1 Transcriptional activation activity analysis of LbMYB48. Yeast cells containing pGBKT7-*LbMYB48* recombinant plasmid were used as the experimental group. Yeast cells containing both pGBKT7-p53 and pGADT7-T vectors were used as the positive control. Yeast cells containing only pGBKT7 empty vector was used as the negative control. These transformed yeast cells were grown onto SDO (SD/-TRP) medium and TDO/X (SD/-Trp/-His/-Ade/X-α-Gal) medium.

Supplementary Figure 2 The relative expression lvels of *LbMYB48* gene in TRV*::LbMYB48* and TRV*::*0 lines. Three replicates should be set up to calculate the standard deviation (SD) and ensure accuracy. Different letters (a–b) were used to indicate significant differences between different columns at *P*< 0.05.

Supplementary Figure 3 Area comparison of other epidermal cells in TRV*::LbMYB48* and TRV*::*0 lines. Three replicates should be set up to calculate the standard deviation (SD) and ensure accuracy.

Supplementary Figure 4 RT-qPCR validation of differentially expressed genes by RNA-seq. Three replicates should be set up to calculate the standard deviation (SD) and ensure accuracy.

## Abbreviations

ABA, abscisic acid; ABI, ABA INSENSITIVE; APX, Aseorbateperoxidase; CRKs, calcium-dependent protein kinase-related kinases; CPC, CAPRICE; cDNA, complementary DNA; DAPI, 4’,6-diamidino-2-phenylindole; DAB, Diaminobenzidine; FPKM, Fragments Per Kilobase of transcript per Million fragments mapped; GLRs, glutamate receptor–like genes; GSTs, Glutathione S-transferases; His, Histidine; H_2_O_2_, hydrogen peroxide; KEGG, Kyoto Encyclopedia of Genes and Genomes; MDA , malondialdehyde; MeJA, Methyl Jasmonate; mRNA, Messenger RNA; NCED, 9-cis-epoxycarotenoid dioxygenase; NBT, nitro-blue tetrazolium; ORF, open reading frame; PAOs, Polyamine oxidases; PHGPx, Phospholipid hydroperoxide glutathione peroxidase; P5CS, Δ-pyrroline-5-carboxylate synthetase; PCR, polymerase chain reaction; K^+^, potassium ion; PP2C, protein phosphatase 2C; RT-qPCR, quantitative Real-time PCR; ROS, reactive oxygen species; RLKs, receptor-like kinases; RNA-seq, RNA-sequencing; SOS, Salt-Overly-Sensitive; TRV, tobacco rattle virus; TFs, transcription factors; Trp, Tryptophan; VIGS, virus induced gene silencing; DIS3, DISTORTED3; DMSO, dimethyl sulfoxide; TCA, trichloroacetic acid; SEM, Scanning electron microscopy; REC, relative electrolytic conductivity; PEG, polyethylene glycol; MS, Murashige and Skoog.
