# Supplementary material for: LbMYB48 positively regulates salt gland development of Limonium bicolor and salt tolerance of plants
Source: Front Plant Sci. 2022 Oct 26;13:1039984. doi: 10.3389/fpls.2022.1039984 (PMC9644043; doi:10.3389/fpls.2022.1039984)
Supplement: Supplementary file 1 [file Table_1.docx]

**Supplementary Table 1** Primer Sequences Used in this Research

| **Different experiments** | **Forward primers** | **Reverse primers** |
| --- | --- | --- |
| Subcellular Localization of LbMYB48 | 5-ATGACTCCTCATGAAGAGAAACTGG-3 | 5-TGGTGCTCCATAGGACGACAG-3 |
| Promoter Sequence of LbMYB48 | 5-TAGATTGTCATACCTTTAACTAT-3 | 5-CTTTCCTCTTTTGAGACTTGG-3 |
| Transcriptional Activation Activity of LbMYB48 | 5-tcagaggaggacctgcatatgATGACTCCTCATGAAGAGAAACTGG-3 | 5-ttcggcctccatggccatatgTCATGGTGCTCCATAGGACGA-3 |
| LbMYB48 Heterologous Overexpression Lines in Arabidospsis | 5-ATGTCTGGGTATCAGCTAGCTAGG-3 | 5-GATCAAGTACTCATAGTACACTAAGAACAACG-3 |
| *RD29A* gene for RT-qPCR | 5-ATCACTTGGCTCCACTGTTGTTC-3 | 5-ACAAAACACACATAAACATCCAAAGT-3 |
| *RD22* gene for RT-qPCR | 5-ATAATCTTTTGACTTTCGATTTTACCG-3 | 5-CTTGGACGTTGGTACTTTTCTCG-3 |
| *AtP5CS1* gene for RT-qPCR | 5-TAGCACCCGAAGAGCCCCAT-3 | 5-TTTCAGTTCCAACGCCAGTAGA-3 |
| *SOS1* gene for RT-qPCR | 5-TTCATCATCCTCACAATGGCTCTAA-3 | 5-CCCTCATCAAGCATCTCCCAGTA-3 |
| *ACTIN2* gene for RT-qPCR | 5-AAGCTGGGGTTTTATGAATGG-3 | 5-TTGTCACACACAAGTGCATCAT-3 |
| *LbTUBULIN* gene for RT-qPCR | 5-GGTTGAGTGAGCAGTTCAC-3 | 5-GATAACCAGCCACACCTTAGC-3 |
| *LbMYB48* gene for RT-qPCR | 5-GCTCAAGAAAGGAAACAA-3 | 5-ACGAGATGGAAGAAGAAC-3 |
| *LbCPC-like* gene for RT-qPCR | 5-GAGTGGGAGTTCATCAATA-3 | 5-ATCGCTCAATCTCTTCTG-3 |
| *LbDIS3* gene for RT-qPCR | 5-GGGAGATAAGAACCAGAA-3 | 5-GACTGTTACCTCTTCCATA-3 |
| *LbSOS3* gene for RT-qPCR | 5-GGAGTCATTGATTATGGG-3 | 5-GTCTCAATAACATCATCTGATA-3 |
| *LbCRK29* gene for RT-qPCR | 5-GATGCTCATGCTCTTATAG-3 | 5-CAGCATAGATTGACTAGC-3 |
| *LbABI5* gene for RT-qPCR | 5-CAGCGATGATGATGAATG-3 | 5-ACCTGTAGCAGTAGTAGTA-3 |
| *LbNCED1* gene for RT-qPCR | 5-TGGTCTTCAAGATTATGATAAG-3 | 5-GATCGAAGCTGGTATAGTA-3 |
| *LbPP2C* gene for RT-qPCR | 5-GAGCTATCAGAGGATCAC-3 | 5-CGTTCGTTAAGGTATCCA-3 |
| *LbAPX3* gene for RT-qPCR | 5-CCGTGAAGATGAAGTATC-3 | 5-GGACAAAGTCTATGGTTG-3 |
| *LbGSTU9* gene for RT-qPCR | 5-CCTTCAATATAATCCCATCC-3 | 5-CCAAGTCTCGTCTATGTA-3 |
| *LbGST* gene for RT-qPCR | 5-GAGTGTATGGAAGCTTGG-3 | 5-GTCTGATAGGTCCATCTTC-3 |
| *LbGLR4* gene for RT-qPCR | 5-CAAGGATAGTGGACTTCA-3 | 5-CCAGAACATTGTTATCAATTG-3 |
