# Supplementary figures and images for: LbMYB48 positively regulates salt gland development of Limonium bicolor and salt tolerance of plants
Source: Front Plant Sci. 2022 Oct 26;13:1039984. doi: 10.3389/fpls.2022.1039984 (PMC9644043; doi:10.3389/fpls.2022.1039984)

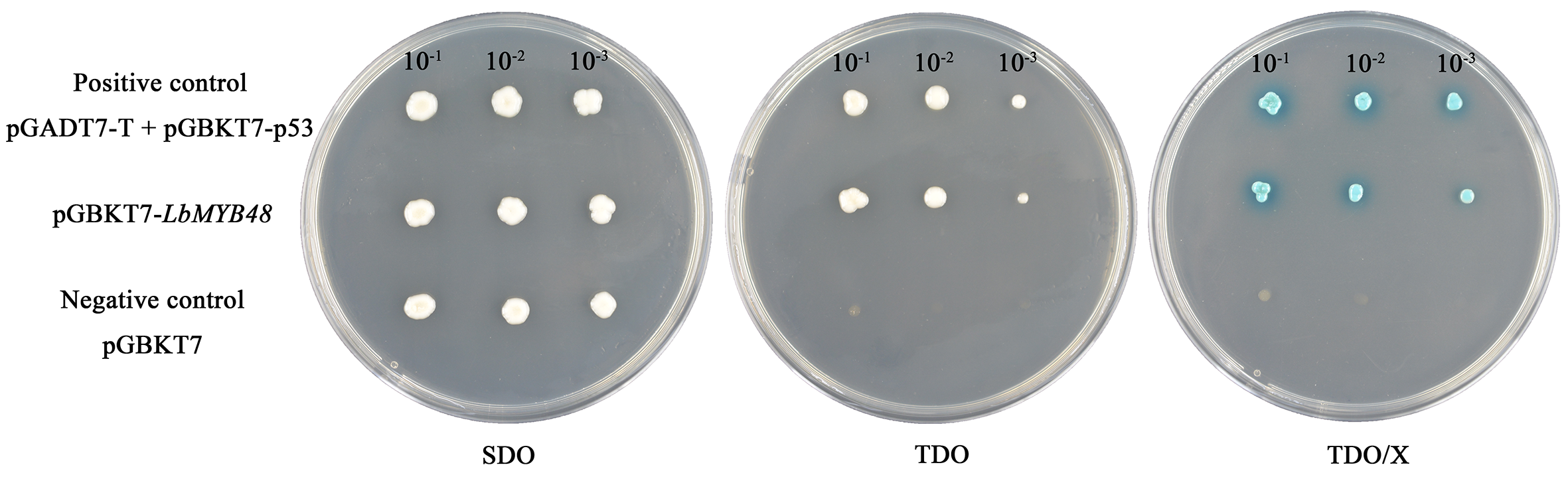

Supplement: Supplementary Figure 1 — Transcriptional activation activity analysis of LbMYB48. Yeast cells containing pGBKT7-LbMYB48 recombinant plasmid were used as the experimental group. Yeast cells containing both pGBKT7-p53 and pGADT7-T vectors were used as the positive control. Yeast cells containing only pGBKT7 empty vector was used as the negative control. These transformed yeast cells were grown onto SDO (SD/-TRP) medium and TDO/X (SD/-Trp/-His/-Ade/X-α-Gal) medium. [file Image_1.tif]

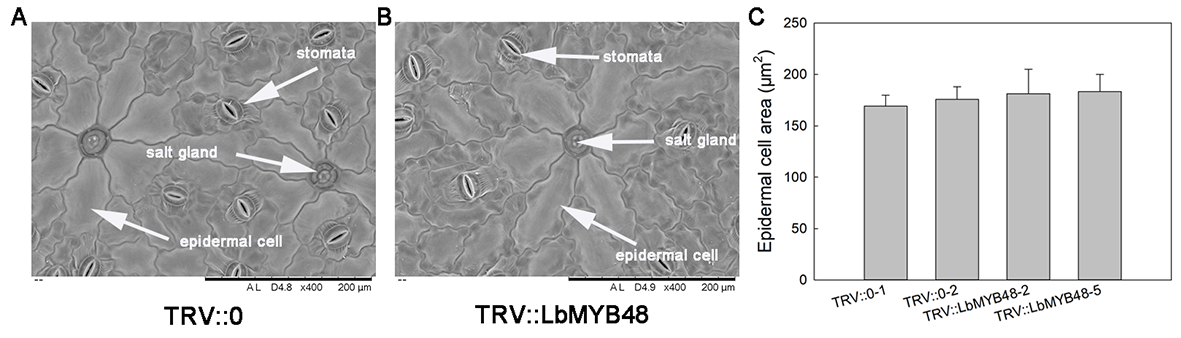

Supplement: Supplementary Figure 3 — Area comparison of other epidermal cells in TRV::LbMYB48 and TRV::0 lines. Three replicates should be set up to calculate the standard deviation (SD) and ensure accuracy. [file Image_3.tif]

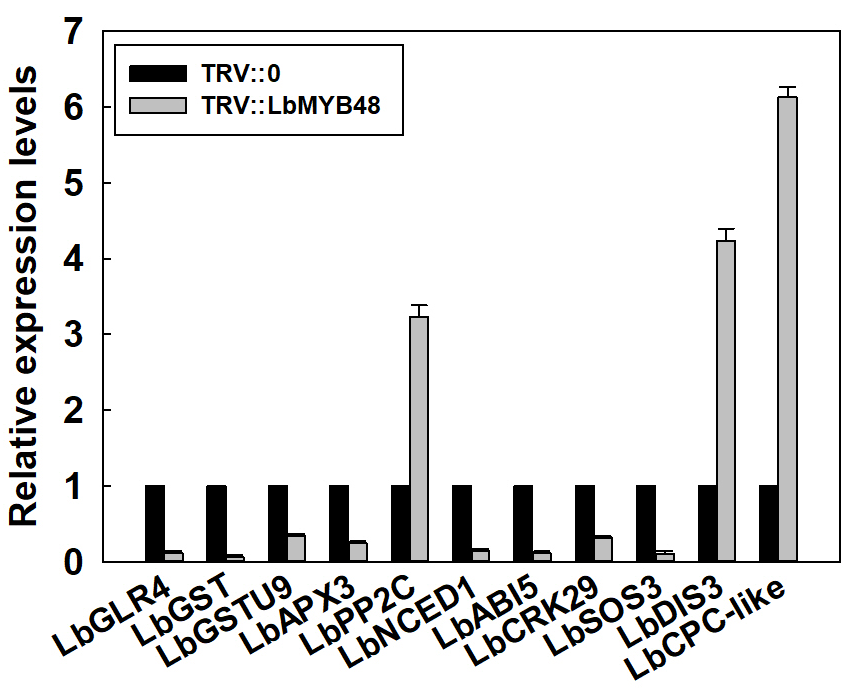

Supplement: Supplementary Figure 4 — RT-qPCR validation of differentially expressed genes by RNA-seq. Three replicates should be set up to calculate the standard deviation (SD) and ensure accuracy. [file Image_4.tif]
